# Diagnostic utility of whole body CT scanning in patients with unexplained weight loss

**DOI:** 10.1371/journal.pone.0200686

**Published:** 2018-07-27

**Authors:** Yonggeng Goh, Yock Young Dan, Wynne Chua, Pooja Jagmohan, Joseph King-Tak Lee, Yee Liang Thian

**Affiliations:** 1 Department of Radiology, National University Hospital, Singapore; 2 Department of Gastroenterology, National University Hospital, Singapore; Weill Cornell Medical College in Qatar, QATAR

## Abstract

**Background:**

Unexplained weight loss is a non-specific complaint with myriad potential etiologies. Increasingly, whole body CT studies are being performed in patients with unexplained weight loss to exclude organic etiologies such as malignancy. Our study aims to assess the diagnostic accuracy and yield of whole body CT in these patients.

**Methods and materials:**

Patients who had a whole body CT scan for investigation of unexplained weight loss as their primary complaint from 2009–2012 were retrospectively reviewed. CT scans were classified into 4 categories: (1) Definite/highly suspicious for underlying organic cause (2) Indeterminate for underlying organic cause (3) No findings accounting for weight loss and only incidental findings and (4) Normal study. Scan findings were correlated with the final diagnosis after all investigations. Univariate logistic regression was performed to determine associations between patient’s baseline variables and positive CT scan findings.

**Results:**

Of 301 eligible patients during the study period, 101 patients were excluded due to known history of malignancy, inadequate follow-up or inadequate scan technique. 200 patients were included in the final analyses. The sensitivity, specificity, positive predictive value, negative predictive value and accuracy of CT for organic pathology were 72.0%, 90.7%, 87.0%, 78.9% and 82.0% respectively. Additional symptoms, abnormal physical examinations, anemia, and raised tumor markers were significantly correlated with positive CT findings. Overall, the diagnostic yield of whole body CT scan for patients with unexplained weight loss was 33.5%.

**Conclusions:**

Whole body CT imaging may be a useful investigation for the noninvasive workup of patients with unexplained weight loss, with diagnostic yield of 33.5% and good sensitivity, specificity, positive and negative predictive values for organic etiologies.

## Introduction

Unexplained weight loss is an important clinical problem associated with increased mortality [1, 2]. The multitude of potential etiologies of weight loss ranging from organic (e.g. infection, malignancy etc.) to nonorganic causes (e.g. psychiatric causes) make it a diagnostic challenge for clinicians. Several observational studies of causes of unexplained weight loss show that up to 60% of patients are found to have organic causes, with malignancy being the most common organic cause [3–5].

Currently, there is no universal agreement on the definition of clinically significant weight loss [6]. The point at which unexplained weight loss becomes a medical concern is hence not certain. Most observational studies and review articles however, accept or define significant weight loss as a 5% or more reduction in body weight over a time period of 6–12 months [4, 7–10].

Investigations with the highest yield in identifying potential organic causes include stool occult blood, gastrointestinal endoscopy, and thyroid function tests [3–6, 11]. Increasingly, clinicians are also utilizing whole body computed tomography (CT) scans for diagnostic workup of the patient presenting with unexplained weight loss to screen for occult malignancy and chronic infection such as tuberculosis [4, 6, 8].

Advances in multidetector CT technology in the past 10 years have improved imaging detail and resolution, which potentially improves its ability to detect malignancy and other organic pathology. The readily availability and reduced cost of CT facilities have lowered the threshold for imaging. Our institutional experience is that more whole body CT studies are being performed for the workup of unexplained weight loss despite the lack of evidence supporting such practice in literature.

Current literature evaluating the utility of whole body CT scans for investigation of patients with unexplained weight loss is surprisingly scarce [6, 12, 13]. To our knowledge, there has only been a single small sample study published by Smith et.al [12]. The study found 8 patients (10.5%) out of a cohort of 67 patients had an identifiable cause of weight loss on CT. Potential disadvantages of whole body CT include radiation exposure, high cost of the scan, and potentially low yield in detecting significant abnormalities in these patients [6].

In this study, we assessed the diagnostic accuracy and yield of whole body CT for detection of organic etiologies in patients presenting with unexplained weight loss. In addition, we sought to identify patient variables associated with positive findings from a whole body CT scan.

## Methods and materials

### Study population

Institutional board approval and waiver for informed consent was obtained for this retrospective study. All patients more than 18 years of age referred to our university hospital’s radiology department for a whole body CT scan (defined as a contrast enhanced CT of the thorax, abdomen and pelvis) from 1^st^ January 2009 to 31^st^ December 2012 for unexplained weight loss were included. Patients with (i) unenhanced or technically inadequate scans (ii) history of previous/known malignancy and (iii) less than 6 months of follow-up after negative investigations were excluded.

### Patient characteristics

A total of 301 patients were referred to our radiology department for a whole body CT scan as an investigation for unexplained weight loss between 1 Jan 2009 and 31 Dec 2012. Of these, 101 patients were excluded: 62 patients with previous history of malignancy or metastatic disease, 9 patients with different imaging protocols (i.e. triphasic or unenhanced scans) and 30 patients with inadequate follow-up (i.e. less than 6 months follow-up with no cause of weight loss established after investigations). 200 patients were included in the final analysis.

The mean age for the remaining 200 patients was 63.9 (range 22–96) years old. 62.5% (n = 125) of the patients were males. The median time of follow-up for these patients was 28.9 months. 62% (n = 124) of patients had documented objective evidence of weight loss (i.e. quantified the amount of weight loss over a unit period) at first presentation. 91/124 patients had weight loss which was significant (defined as 5% or more reduction in body weight over a time period of 6–12 months). (Table 1)

**Table 1:** Baseline characteristics of patients (n = 200).

Characteristic	Number
**Age(yrs)–mean(age-range)**	63.9 (22–96)
- >65 yrs–no. (%)	101 (50.5%)
- ≤ 65 yrs–no, (%)	99 (49.5%)
**Male Sex–no. (%)**	125 (62.5%)
**Referring Specialty–no. (%)**	
- Gastroenterology	96 (48%)
- General Medicine	38 (19%)
- Respiratory	20 (10%)
- Geriatrics	7 (3.5%)
- Others (e.g. Infectious disease, Gynecology, Orthopaedics, Hematology, Rheumatology, Cardiology etc.)	39 (19.5%)
**No. of patients presenting with objective amount of weight loss–no. (%)**	124 (62%)
- >5% loss in body weight over 6–12 months	91/124

### CT image acquisition

All patients underwent contrast-enhanced CT imaging acquired with 64- or 128- multidetector row CT of the chest, abdomen and pelvis. Intravenous administration of iohexol (300mg iodine/ml, Omnipaque 300) at a dose of 2 mls/kg body weight was injected via power injector at a flow rate appropriate to the cannula size (3ml/s for 20G and 2ml/s for 22G). Portal venous phase imaging was performed in a craniocaudal direction with aid of bolus-tracking, typically after 65–70 second delay (parameters: 120kVp, 170–350 mAs; collimation, 0.6 mm). Routine dataset reconstructions at 5.0 mm section thickness were performed in axial and coronal planes.

### Data collection

Data including age, sex, accompanying symptoms, physical examination and referring department were retrieved from electronic medical records. Results of baseline evaluation for all patients including biochemical studies (baseline blood tests: complete blood count, CRP, ESR, liver function tests, thyroid function and tumor markers) and radiological investigations (e.g. CXR, ultrasound) and additional investigations where available (e.g. endoscopy) were recorded. The CT studies for these patients were interpreted and prospectively reported by consultants specialized in body imaging. The CT reports were retrieved from an electronic database and reviewed retrospectively.

**Fig 1:**
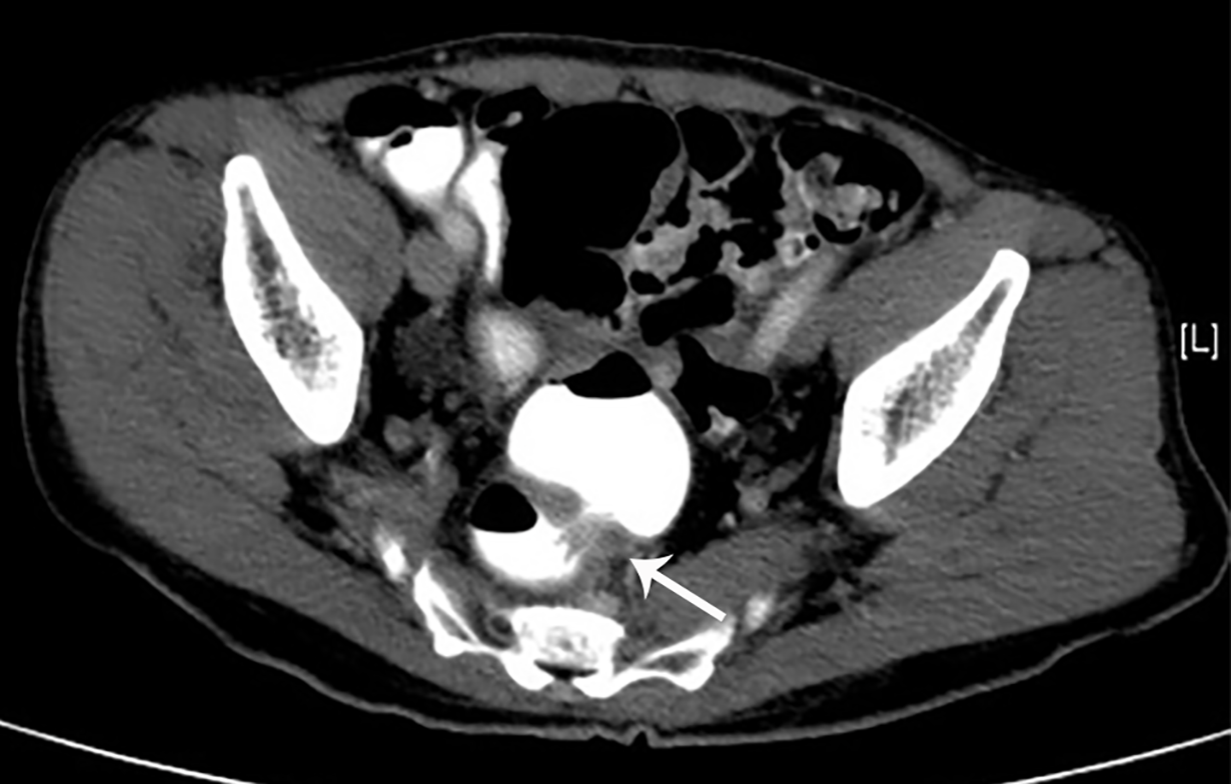
False positive category 2 scan–findings indeterminate for possible organic etiology and require further workup. Axial CT scan of a 74 year old male which demonstrates wall thickening of the rectosigmoid junction (arrow). Follow-up colonoscopy demonstrated no suspicious lesion at the rectosigmoid junction.

**Fig 2:**
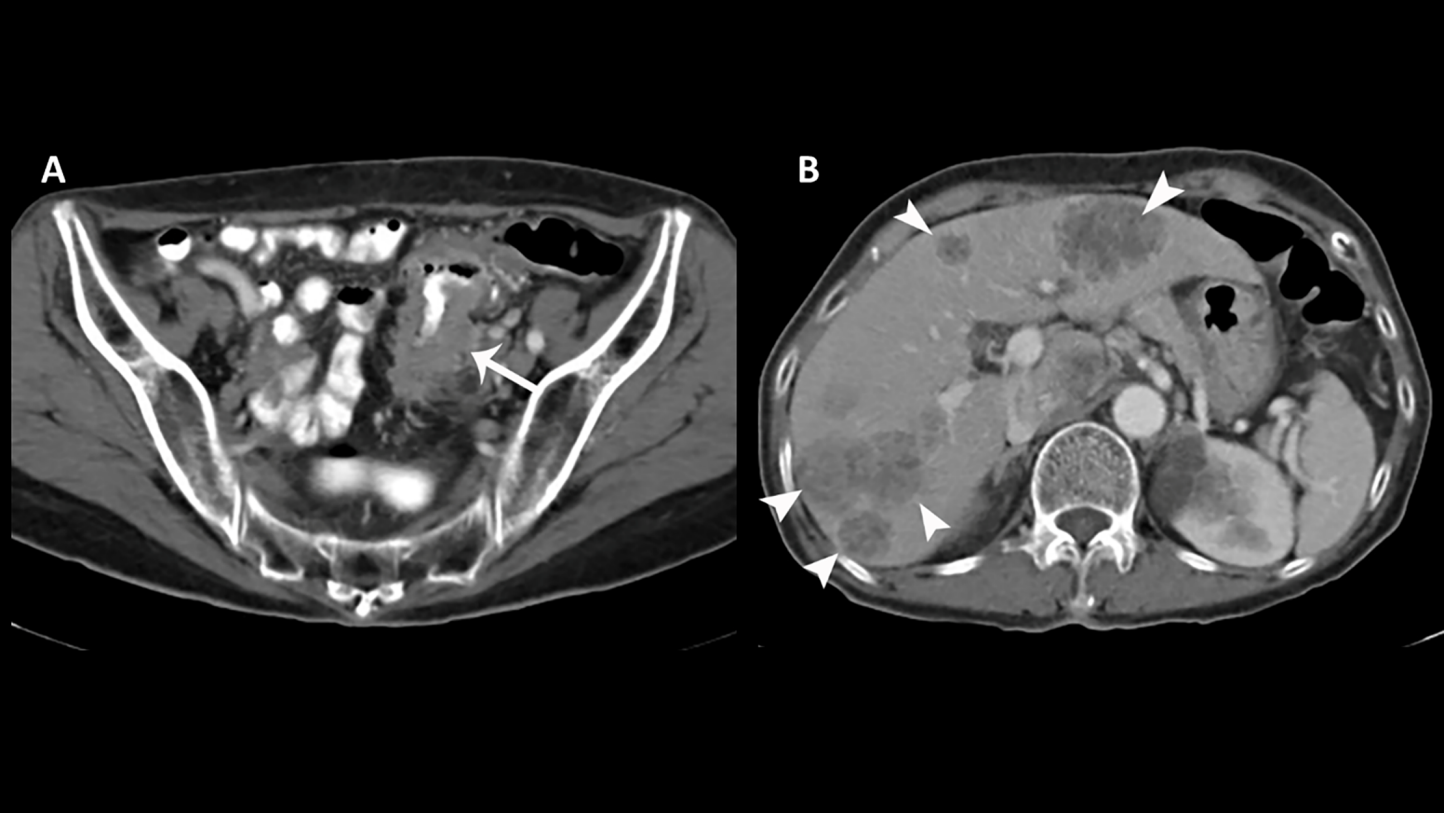
True positive category 1 scan—findings highly suspicious for organic etiology. (A) Axial CT scan of an 80 year old male demonstrates bowel wall thickening of the sigmoid colon (arrow) suspicious for underlying malignancy. (B) Multiple hypodense hepatic lesions are consistent with metastases (arrowheads).

CT scan reports were classified into 4 categories: (1) Definite/highly suspicious for underlying malignancy or organic cause directly accounting for weight loss (Fig 1), (2) Indeterminate for underlying malignancy/organic cause which require further investigation (Fig 2). These included: bowel wall thickening, pulmonary nodules ≥7mm, liver cirrhosis, hepatosplenomegaly, polyps, adrenal nodules, uterine/cervical enlargement and ovarian cysts, (3) No findings accounting for weight loss and only incidental findings of low clinical significance not requiring further workup and (4) Normal study. Positive CT scans were defined as categories 1 or 2.

Follow-up was performed through 15 December 2015 by reviewing the electronic medical records. The details obtained from these clinical entries included last follow-up date and final diagnosis for weight loss etiology which was based on the clinician’s assessment of all available patient biochemical, radiological, endoscopic, surgical findings and psychiatric evaluations where appropriate. The diagnoses of patients were established in 59 patients via biopsy/surgery, 16 patients via imaging, 4 patients via endoscopy and 121 patients via a combination of laboratory tests and clinical follow-up. Patients who had no organic diagnosis recorded were followed up for a minimum period of 6 months to confirm no clinical deterioration.

### Statistical methods

All statistical analyses were performed with Stata 12 (StataCorp. 2011. *Stata Statistical Software*: *Release 12*. College Station, TX: StataCorp LP). Descriptive statistics were used to examine the baseline characteristics of the included subjects. Diagnostic yield was defined as the number of subjects with true positive CT findings for organic etiologies relative to all subjects. The sensitivity, specificity, positive and negative predictive value of CT for detection of organic etiology in patients were also calculated. Simple logistic regression was applied to correlate dichotomous baseline patient variables with positive CT scan findings. All statistical tests were two-sided and a p value of < 0.05 was considered significant.

## Results

### CT findings

CT findings were categorized into four groups (see Methods).

In group 1, CT demonstrated findings highly suspicious for malignancy or an organic etiology of weight loss. 56 patients (28%) were in this group and all patients had an organic cause of weight loss identified on CT which was subsequently confirmed on follow-up and further investigations (e.g. biopsy)

In group 2, CT demonstrated indeterminate findings which may represent an underlying malignancy/organic etiology for weight loss and which needed further workup. 21 patients (10.5%) were in this group. Of these 21 patients, the CT finding was subsequently established as directly related to the cause of weight loss in 11 patients (48%). There were 10 false positive findings: 6 due to suspected bowel wall thickening which was normal on follow-up endoscopy, 2 adrenal nodules which were subsequently classified as incidental and 2 cases of suspected uterine/cervical enlargement which were subsequently found to be normal at gynaecological review.

In group 3, CT demonstrated no findings suggestive of underlying malignancy/organic etiology and only incidental findings of low clinical significance. 80 patients (40%) were in this group. Incidental findings included chronic lung conditions (e.g. fibrosis, old TB changes), pulmonary nodules <7mm, renal/hepatic cysts, benign liver lesions (e.g. hemangiomas), gallstones, diverticular disease and fibroids. There was no organic pathology found in 66/80 patients upon subsequent clinical investigations and follow-up. 14 patients subsequently were diagnosed with an organic disorder. These were sigmoid cancer (1), non-malignant gastrointestinal disorders (7), autoimmune disorders (3), neurological disorders (2) and endocrine disorders (e.g. diabetes, hyperthyroidism) (1).

In group 4, CT was normal. 43 patients (21.5%) were in this group. In 31/43 patients, subsequent clinical investigations and follow-up did not demonstrate organic pathology or clinical deterioration. 12 patients subsequently had an organic disorder diagnosed. These were rectal carcinoma (1), non-malignant gastrointestinal disorders (5), neurological disorders (1), autoimmune disorders (2), infectious disorder (1) and medical disorders (2).

The results are summarized in Table 2.

**Table 2:** Classification of CT Findings in 200 patients with unexplained weight loss.

	No. of pts	Category	Conditions listed in group	No. of patients with organic causes confirmed	PPV (95% CI)	NPV (95% CI)
**Group 1**	56 (28%)	Highly suspicious for underlying malignancy/organic cause	Malignancies, Occult Sepsis, Significant lymphadenopathy	56	100% (93.62–100%)	NA
**Group 2**	21 (10.5%)	Indeterminate for underlying malignancy/organic cause	Bowel wall thickening, Pulmonary nodules ≥7mm, Liver cirrhosis, Hepatosplenomegaly, GI polyps, Adrenal nodules, Uterus/Cervix enlargement, Ovarian cysts of indeterminate appearance	11	52.4% (29.8–74.3%)	NA
**Group 3**	80 (40%)	Incidental findings of low clinical significance	Chronic lung conditions (e.g. fibrosis, chronic TB), Pulmonary nodules <7mm, Renal cysts, Hepatic cysts, Benign liver lesions (e.g. hemangioma), Gallstones, Diverticular disease, Fibroids	14	NA	82.5% (72.4–90.1%)
**Group 4**	43 (21.5%)	Normal study	Not applicable	12	NA	72.1% (56.3–84.7%)

Overall, CT demonstrated positive findings in 38.5% of patients in our cohort. The sensitivity, specificity, positive predictive value, and negative predictive value of CT for detection of organic etiology in patients presenting with unexplained weight loss were as follows: 72.0 (61.2–80.1) %, 90.7 (83.5–95.4) %, 87.0 (77.4–93.6) %, and 78.9 (70.6–85.7) %. The sensitivity, specificity, positive predictive value, and negative predictive value of CT for detection of malignant etiology were as follows: 95% (84.5–99.4%), 77.6% (70.2–83.9%), 54.6% (42.8–65.9%) and 98.4% (94.2–99.8%). Overall, the diagnostic yield (true positive rate) of whole body CT scan for patients with unexplained weight loss was 33.5%.

### Causes of unexplained weight loss

The cause of unexplained weight loss was established in 61.5% (123/200) of patients after all investigations. The causes are summarized in Table 3. Overall, the three most common organic causes of unexplained weight loss were malignancy (22%), infections (9%) and non-malignant gastrointestinal disorders (6.5%). The cause of weight loss in remaining 77 patients was still unknown in spite of extensive investigations.

**Table 3:** Causes of weight loss.

Diagnosis	Number	Positive findings on CT
Malignancy	Total: 44	
Gastrointestinal tract	20	18
Respiratory tract	11	11
Hematological	5	5
Gynecological	4	4
Hepatobiliary	2	2
Urinary Tract	1	1
Thyroid	1	1
Non-malignant organic disorders	Total: 48	
Infectious disorders (e.g. HIV, TB)	18	17
Gastrointestinal disorders (e.g. inflammatory bowel disease)	13	1
Gynecological disorders	2	2
Autoimmune disorders (e.g. rheumatoid arthritis)	9	4
Neurological disorders (e.g. Parkinson’s)	3	0
Medical disorders (e.g. hyperthyroidism, diabetes)	3	0
Psychiatric disorders	Total: 18	
Depression	7	0
Anxiety/Panic disorders	7	0
Aging/Dementia	4	0
Dietary causes	Total: 12	1
Side effects from medications	Total: 1	0
No cause identified	Total: 77	

### Association between patient baseline variables and positive CT findings

Associations between positive CT scan findings and other baseline variables assessed by univariate logistic regression analysis are shown in Table 4. The presence of additional symptoms, abnormal physical examination, anemia and elevated tumor markers were significantly associated with positive (group 1 or 2) CT scan findings. 1/30 patients (3.3%) who had a normal baseline clinical evaluation (normal physical examination and normal blood tests) had a significant positive finding on CT scan.

**Table 4:** Association between positive CT scan findings and baseline patient variables using univariate logistic regression.

Factor Tested	Odds Ratio (95% CI)	P-value
Elderly age (>65 years)	0.75 (0.42–1.33)	0.327
Sex (Male)	0.70 (0.39–1.26)	0.234
Additional symptoms	2.63 (1.27–5.42)	0.009
Abnormal physical examination	3.25 (1.67–6.35)	0.001
Anemia	2.14 (1.13–4.05)	0.019
Abnormal total white cell count	2.20 (0.97–4.87)	0.051
Elevated tumor markers	10.7 (2.88–39.9)	<0.001

## Discussion

Our study indicated that a whole body CT scan may be a useful investigation in the diagnostic workup of patients with unexplained weight loss, with diagnostic yield of 33.5%, and high positive and negative predictive values for all organic etiologies of 87% and 79% respectively. In particular, whole body CT was sensitive for detection of malignancy and chronic infection; positive CT scan findings were found in 95% (59/62) of patients eventually diagnosed with malignant or infective etiologies for weight loss.

A whole body CT had a high negative predictive value for malignancy in our cohort [NPV = 98.4% (94.2–99.8%)]. Among patients who had a normal or near normal CT, only 1.6% (2/123) of patients were diagnosed with malignancy after further investigation and follow-up. Both patients with falsely negative scans had colorectal carcinoma. These false negative cases are attributable to the known limited sensitivity of CT for colonic lesions when there is poor distension of bowel loops and inadequate bowel cleansing preparation [14, 15]. It is possible that a whole body CT incorporating CT colonography protocol to improve detection of colonic lesions could further increase the scan sensitivity [16].

We found CT imaging was less useful for detection of abnormalities secondary to non-malignant gastrointestinal disorders. In this group, CT was only able to detect abnormality in 7.7% of patients (1/13). Hence, gastrointestinal endoscopy is still essential for diagnosis in this group of patients. CT also had poor sensitivity for autoimmune, neurological and medical disorders in our cohort. This is not surprising given that our whole body CT protocols do not include coverage of the brain, and most medical disorders may not result in morphological changes detectable on CT scan unless there was end organ damage. Interestingly, 4/9 patients with autoimmune conditions had positive CT scan findings due to abnormal CT findings (3 patients had significant lymphadenopathy and 1 patient had morphological features of liver cirrhosis) which necessitated further investigation.

Published literature on the utility of whole body CT imaging for patients presenting with unexplained weight loss is scarce. The authors were able to find only a single small sample study by Smith et.al [12]. Smith found positive CT scan findings accounting for weight loss in 10.5% (8/67) of their cohort, in contrast to 33.5% (67/200) in our study. Our higher incidence of positive scan findings may be due to differences in patient referral patterns, differences in clinician ordering thresholds for whole body CT scans, and geographic differences in disease prevalence (possibly higher prevalence of infectious diseases such as tuberculosis in our area of practice).

In our study, several patient variables were significantly associated with a positive finding (Group 1 or 2) on CT. These included additional localising or systemic symptoms, abnormal physical examination, anemia and elevated tumor markers. We also found that only 3.3% (1/30) of our cohort who had a normal baseline clinical evaluation (normal physical examination and normal blood tests) had a significant positive finding on whole body CT. This corroborates recommendations from other authors that a comprehensive history, physical examination and basic blood tests should be the first step in assessing the need for further investigation and imaging [4, 6].

The utility of whole body CT screening has been studied in other clinical populations such as in asymptomatic patients [17] and patients with deep vein thrombosis [18]. These studies have demonstrated low yields for clinically significant findings. Among 1192 asymptomatic patients studied by Furtado et al. only 4.8% had clinically significant abnormalities [17]. In the study by Carrier. et al [18] on patients with unprovoked venous thromboembolism, 4.7% (19/423) of their cohort were diagnosed with occult malignancy. In contrast, whole body CT screening had a diagnostic yield of 33.5% in our cohort, with malignant causes found in 21%. The higher diagnostic yield likely reflects higher pre-test probability in patients with unexplained weight loss as compared to other clinical scenarios. The prevalence of malignancy in our cohort is concordant with previously published studies on etiologies of unexplained weight loss [3–5, 8, 9, 13].

CT imaging has been shown to yield a high prevalence of incidental findings unrelated to the primary complaint such as hepatic cysts, renal cysts, small thyroid nodules, and small pulmonary nodules [6, 17]. In our study, a large proportion (40%) of patients had such incidental findings on whole-body CT. Although the vast majority of these incidental findings would be benign and of no clinical significance, many physicians and patients are unwilling to accept uncertainty even when the chance of a serious diagnosis is extremely unlikely. Thus their discovery may provoke anxiety, lead to a cascade of testing and follow-up that is costly, and may even cause morbidity from unnecessary biopsies. However, a detailed evaluation of the impact of these incidental findings and cost-benefit analysis were beyond the scope of this study. Other drawbacks of a whole body CT include the high cost of the procedure, high radiation dose exposure, and potential for allergic reactions and renal impairment as a consequence of the contrast medium administered during the procedure. Therefore, the potential risks of whole body CT, high prevalence of incidental findings, and possibly negative yield should be discussed with patients prior to whole body CT imaging as part of informed consent.

Our study has several limitations. First, we lacked a single diagnostic gold standard to determine the final diagnosis of patients included in our study, but this is due to the multitude of possible etiologies for weight loss. We used a combination of clinical examination, biochemical tests, radiological studies, histology, endoscopy and clinical follow-up of a minimum of 6 months duration as diagnostic end-points. It is possible that a longer period of follow-up and more extensive testing may yield additional organic diagnosis but it has been found that further extensive testing has low yield [4]. The prevalence of organic etiologies in our cohort (46%) is notably concordant with that reported in literature among patients with unexplained weight loss. Secondly, weight loss in our cohort was defined by the patient, with only 62% having objective documented evidence of weight loss. However, all patients in our study presented with a history of substantial weight loss which the clinician felt warranted formal workup and thus our cohort reflects actual clinical practice and referral patterns. Thirdly, the referrals for whole body CT imaging came from different clinicians with likely different thresholds for imaging. Fourth, the timing of CT scans in relation to the work-up of unexplained weight loss was not factored in our study and would have been variable as this was a retrospective study. Lastly, this was a retrospective study, and our findings require validation with a prospective patient cohort.

## Conclusion

Whole body CT imaging may be a useful investigation for the noninvasive workup of patients with unexplained weight loss, with diagnostic yield of 33.5% and good sensitivity, specificity, positive and negative predictive values for organic etiologies. Patients with additional symptoms, abnormal physical examination, anemia and raised tumor markers are significantly more likely to have positive findings on CT. This group of patients may derive the most diagnostic value from whole body CT imaging.

## Supporting information

S1 File This is the anonymized data set of our 200 patients collected. Attached within the file are the signs, symptoms, blood test abnormalities, CT findings and eventual diagnosis of the 200 patients.
(XLS)
